# A new dry eye mouse model produced by exorbital and intraorbital lacrimal gland excision

**DOI:** 10.1038/s41598-018-19578-6

**Published:** 2018-01-24

**Authors:** Katsuhiko Shinomiya, Mayumi Ueta, Shigeru Kinoshita

**Affiliations:** 1 Department of Ophthalmology, Kyoto Prefectural University of Medicine, Kyoto, Japan; 2 Pharmacology Group, Non-Clinical Research, Global R&D Division, Santen Pharmaceutical Co., Ltd., Ikoma, Japan; 3 Department of Frontier Medical Science and Technology for Ophthalmology, Kyoto Prefectural University of Medicine, Kyoto, Japan

## Abstract

Chronic dry eye is an increasingly prevalent condition worldwide, with resulting loss of visual function and quality of life. Relevant, repeatable, and stable animal models of dry eye are still needed. We have developed an improved surgical mouse model for dry eye based on severe aqueous fluid deficiency, by excising both the exorbital and intraorbital lacrimal glands (ELG and ILG, respectively) of mice. After ELG plus ILG excision, dry eye symptoms were evaluated using fluorescein infiltration observation, tear production measurement, and histological evaluation of ocular surface. Tear production in the model mice was significantly decreased compared with the controls. The corneal fluorescein infiltration score of the model mice was also significantly increased compared with the controls. Histological examination revealed significant severe inflammatory changes in the cornea, conjunctiva or meibomian glands of the model mice after surgery. In the observation of LysM-eGFP^(+/−)^ mice tissues, postsurgical infiltration of green fluorescent neutrophils was observed in the ocular surface tissues. We theorize that the inflammatory changes on the ocular surface of this model were induced secondarily by persistent severe tear reduction. The mouse model will be useful for investigations of both pathophysiology as well as new therapies for tear-volume-reduction type dry eye.

## Introduction

Dry eye disease is defined as a chronic and multifactorial disorder of the ocular surface epithelium and its accompanying tear film which results in ocular discomfort and visual impairment^1^. It is known that the popularization of air conditioning^2^, frequent usage of visual display terminals (VDT)^3–5^ such as those used with personal computers and smartphones, and an increase in wearers of soft contact lenses^5–8^ all may be associated with a rise in the incidence of dry eye. Currently, dry eye disease is the most common eye disease, affecting more than 5% of the world’s population, with higher prevalence in postmenopausal women (6–9.8%)^9,10^. In Japan, the number of dry eye patients is also increasing, and more than 15% of the population (about 20,000,000 persons) may have at least one symptom of dry eye^5^. The ocular dryness and discomfort associated with dry eye disease can progress to visual disturbance and instability of the tear film, potentially leading to damage to the ocular surface. It may have an impact on the ability to work or read and to drive at night^11,12^. Moreover, it is known that some dry eye patients have chronic pain. Previous studies suggest that neuropathic pain may be associated with chronic pain of dry eye^13^. Therefore, the quality of life of dry eye patients is notably lower when compared to the normal expectation. Disruption of mucosal immune tolerance drives disease progression in an animal model of dry eye^14,15^, suggesting that dry eye may be also an autoimmune disorder.

It is well known that severe dry eye is a signature pathophysiological feature of both Sjögren syndrome (SS) and Stevens-Johnson syndrome (SJS). SS is a systemic autoimmune disease, in which lymphocytic infiltration of salivary and lacrimal glands leads to immune-mediated secretory dysfunction^16^. SS patients often show severe aqueous deficiency, with dry eye being the result of lacrimal gland dysfunction. SJS and its most severe pathophysiological manifestation, toxic epidermal necrosis (TEN), are acute inflammatory vesiculobullous reactions of the skin and mucosa, including the ocular surface, oral cavity, and genitals^17^. SJS/TEN is a side effect of various drugs^18–20^, and we previously reported that SJS/TEN with severe ocular complications such as severe dry eye is greatly associated with use of common cold medicines such as nonsteroidal anti-inflammatory drugs and acetaminophen^18,19^. It is also known that the pathophysiology of dry eye accompanying these disorders is caused by a severe deficiency in hydration of the ocular surface.

For a better understanding of the pathogenesis of, or the molecular mechanisms contributing to, dry eye disease, especially severe aqueous deficiency dry eye caused by SJS/TEN, the appropriate animal model is necessary. Such an animal model also would be useful in the research and development of new ocular medicines and devices. Many kinds of experimental animal models of dry eye disease have been reported previously, including those utilizing monkey^21^, rabbit^22,23^, and rat^24^. Several genetically modified or spontaneous mouse models have been proposed for dry eye diseases including Sjögren’s syndrome^25,26^. However, we considered that these previously reported animal models for dry eye may not sufficiently mimic severe aqueous deficiency at the ocular surface or may in some cases be problematic to carry out in practice.

**Figure 1:**
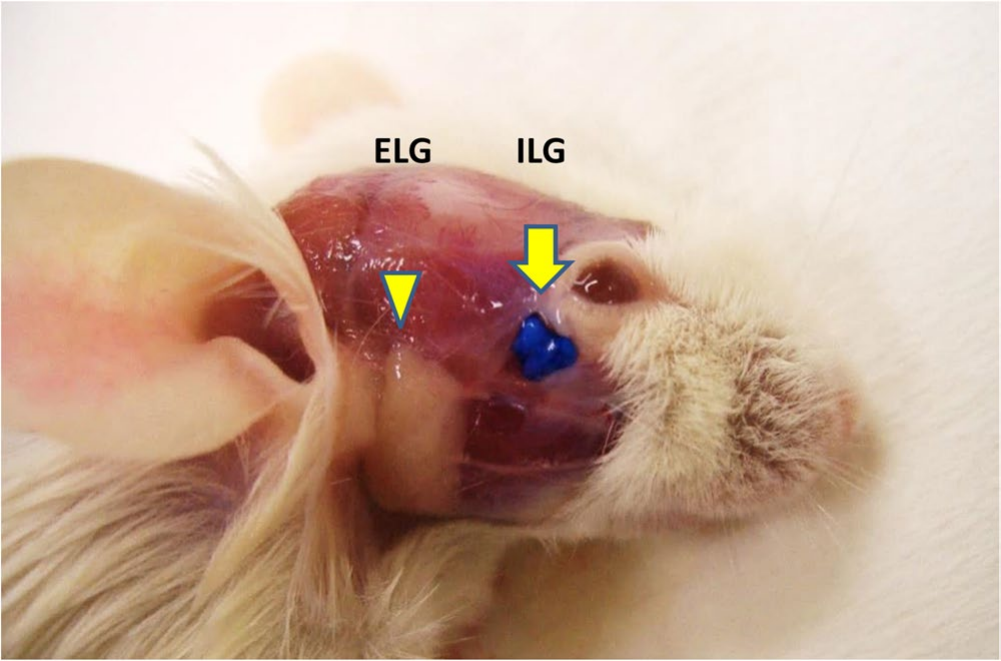
Location of the murine ELG and ILG (Balb/c mouse). Cephalic epidermis and subcutaneous tissue were removed after sacrifice. The ILG was colored blue by painting with a dye (arrow). The ELG (arrowhead) is larger than the ILG. Photograph courtesy of Shibata *et al*. (R&D Division, Santen Pharmaceutical Co., Ltd.).

It is well known that the main role of the lacrimal gland in many animals is tear secretion, and several dry eye animal models have been developed by means of lacrimal gland excision^21,23,24^. In rodents such as mice or rats, the lacrimal gland exists as two anatomically distinct divisions. The exorbital lacrimal gland (ELG) is the main lacrimal gland and in an adult mouse is located subcutaneously approximately 3 mm to the temporal side of the eye, anterior to the ear. By comparison, the intraorbital lacrimal gland (ILG) is considerably smaller than the ELG and is located under the bulbar conjunctiva of the outer canthus (Fig. 1)^27,28^. The joint excretory duct of both ipsilateral ILGs opens at this outer canthus^29^. Rodents as well as many other vertebrates also have another secretary gland called the Harderian gland (HG), located in the deep part of the orbit, and which mainly secretes lipids and porphyrins^29,30^, though it is thought that the HG has a different role compared with the lacrimal gland. In rat, an ELG excision dry eye model has been previously reported^24^, and tear volume in this model was approximately half that of non-treated control. Recently, another group published a report regarding the murine ELG excision model^31^, and in this case, the ELG could be excised simply, resulting in increased ocular surface damage, detectable by fluorescein staining, as well as persistent tear volume reduction. However, according to our original evaluation, histological changes observed in the ELG-only excision model, such as inflammatory cell infiltration of ocular surface tissues, were not sufficiently pronounced (Shinomiya K *et al*., ARVO 2014 annual meeting, May 4, 2014). With the goal of producing a more relevant animal model for severe dry eye, in effect simulating SS or SJS/TEN, we reasoned that it was necessary to replicate the changes occurring at the ocular surface by means of extreme tear fluid reduction. Based on our preliminary results, we theorized that the tear formation decrease of the ELG-excised mouse might be made more severe by additionally excising the ILG. However, it was our determination that the commonly employed surgical technique for ILG excision in the mouse was difficult to perform reproducibly and without complications, because the tissue was very thin and was located anatomically at the deeper part of the orbit containing many blood vessels^27^. In this report we demonstrate the ophthalmological and histological characteristics, and also the preclinical research utility, of a severe aqueous-deficient dry eye model, the ELG plus ILG excised dry eye model mouse, by comparing it with the ELG-only excision model. Moreover, we also have devised a simple method to excise the ILG by approaching it via the subcutaneous tissue of the eye’s temporal lid margin.

## Results

### Tear production in the ELG plus ILG excised mice was significantly decreased

**Figure 2:**
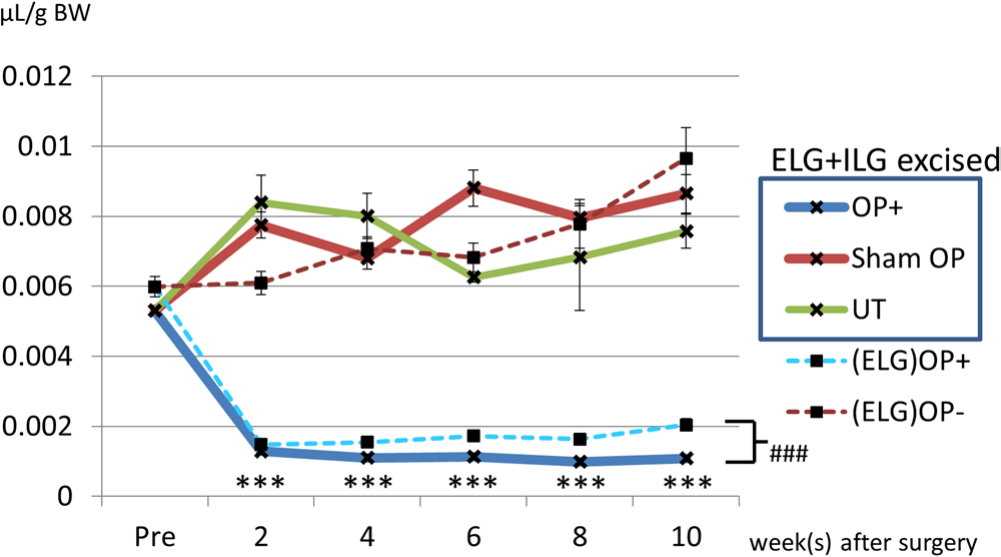
Change of tear volume in the ELG plus ILG excised mice and ELG excised mice. The graph showed the change of tear volume in the ELG plus ILG excised mice (OP+; blue line), sham operated mice (Sham OP; red line) and untreated mice (UT; green line) at 2, 4, 6, 8 and 10 weeks after surgery, as well as before surgery. For comparison, the data of ELG-only excised mice ((ELG) OP+; dotted blue line) and untreated controls ((ELG) OP-; dotted red line) were overlaid. Tear production in the ELG plus ILG excised mice (OP+; blue line) was significantly decreased compared with those receiving the sham operation (Sham OP; red line), or with untreated controls (UT; green line). The reduction of tear volume was continuously observed for 10 weeks after surgery. X-axis shows time point after surgery. Y-axis shows calculated tear volume per 1 g of animal’s body weight (BW), using a standard curve. In addition, tear production in the ELG plus ILG excised mice was significantly decreased compared with the ELG only excised mice ((ELG) OP+; dotted blue line). Each data point represents mean ± SEM. *** indicates statistical significance (P < 0.001) compared with sham OP by unpaired Student’s t-test or Welch’s t-test. ### indicates statistical significance (P < 0.001) between ELG plus ILG excised mice and ELG-only excised mice by unpaired Student’s t-test.

Tear production in the ELG plus ILG excised mice was significantly decreased compared with the sham surgery and untreated controls beginning at 2-weeks after surgery (Fig. 2). In addition, the reduction of tear volume in the ELG plus ILG excised mice was continuously observed for 10-weeks after surgery without evident recovery. Tear production in the ELG plus ILG excised mice eventually was reduced to 16.6%, 16.2%, 12.4%, and 12.5% of the mean value assessed for sham surgery controls at 2-, 4-, 8-, and 10-weeks after surgery, respectively. Compared with the ELG-only excision model (our preliminary study, unpublished data), a more pronounced reduction, approximately by 53% in tear volume was observed at 10-weeks after surgery (Fig. 2).

### The corneal fluorescein infiltration score of the ELG plus ILG excised mice was highly elevated

**Figure 3:**
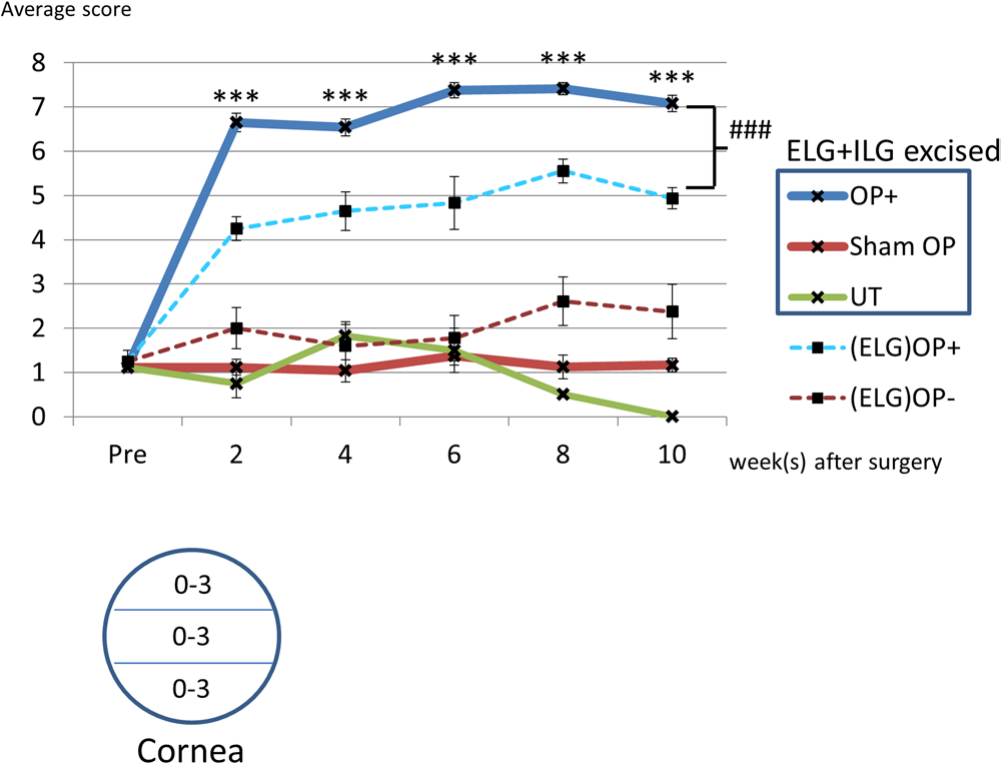
Change in the fluorescein infiltration scores for ELG plus ILG excised mice and ELG excised mice. The graph showed the change of fluorescein infiltration score in the ELG plus ILG excised mice (OP+; blue line), sham operated mice (Sham OP; red line) and untreated mice (UT; green line) at 2, 4, 6, 8 and 10 weeks after surgery, as well as before surgery. For comparison, the data of ELG-only excised mice ((ELG) OP+; dotted blue line) and untreated controls ((ELG) OP-; dotted red line) were overlaid. The corneal fluorescein infiltration scores for ELG plus ILG excised mice (OP+; blue line) were significantly increased compared with the sham operation (Sham OP; red line) and untreated controls (UT; green line). In addition, the fluorescein infiltration score of the ELG plus ILG excised mice (OP+; blue line) was significantly increased compared with the ELG-only excised mice ((ELG) OP+; dotted blue line) at all time points. The corneal fluorescein infiltration score was assigned at each 1/3 area of the cornea (upper, intermediate, and lower) as shown in the depicted scheme. The score was categorized from 0 to 3 (0 = no fluorescence, 1 = fluorescence resembling sparse dots, 2 = dense dot-like pattern, 3 = very dense dot-like fluorescence) depending on the observation. The infiltration pattern between 0 and 1, 1 and 2, 2 and 3 were categorized as 0.5, 1.5, or 2.5, respectively. Each data point represents mean ± SEM. *** indicates statistical significance (P < 0.001) compared with sham OP by unpaired Student’s t-test or Welch’s t-test. ### indicates statistical significance (P < 0.001) between ELG plus ILG excised mice and ELG excised mice by unpaired Student’s t-test.

The fluorescein infiltration score of the ELG plus ILG excised mice was significantly increased compared with the sham surgery and untreated controls (Fig. 3).

**Figure 4:**
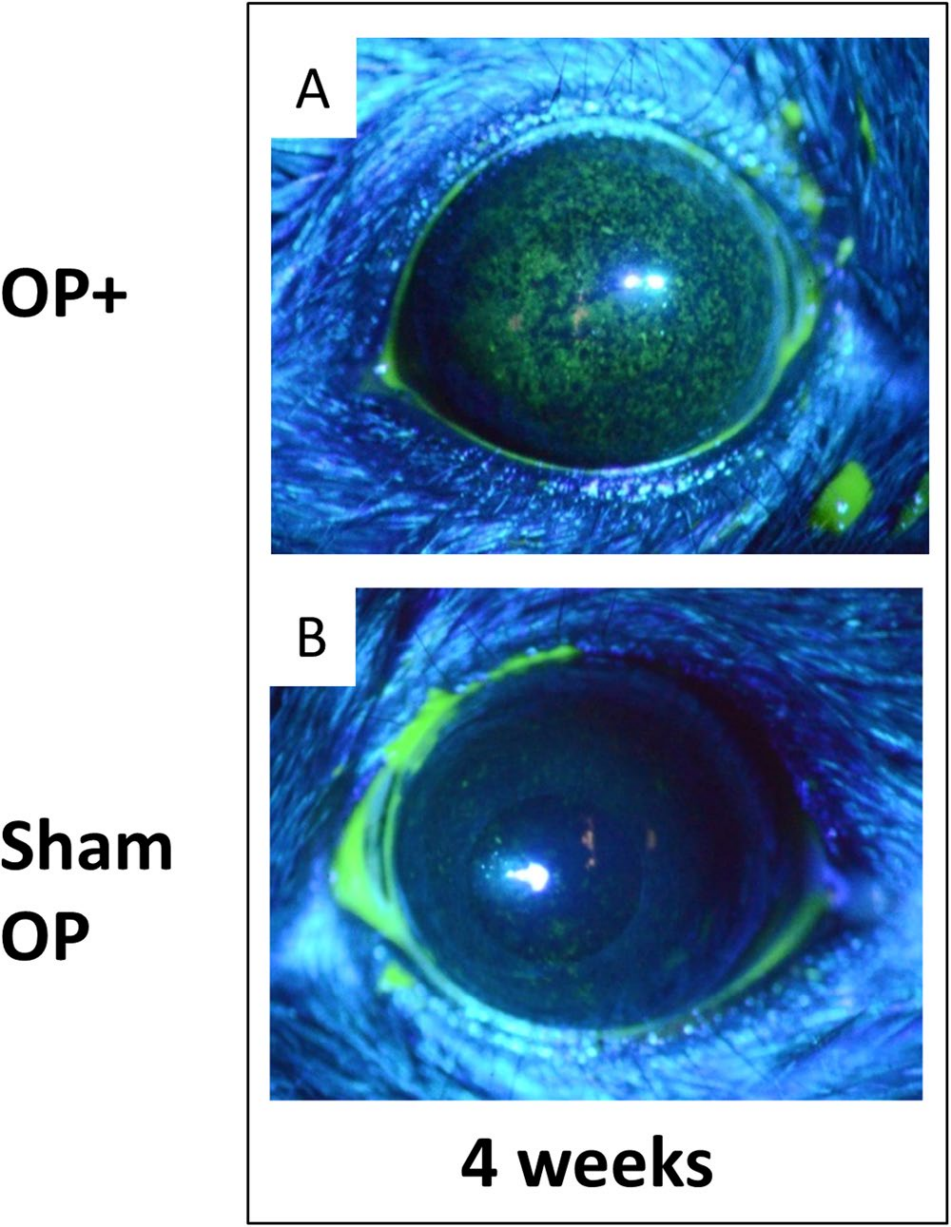
Corneal fluorescein infiltration at 4-weeks after surgery in the ELG plus ILG excised mice. Starry sky-like dense SPK was observed in the ELG plus ILG excised eye at 4 weeks after surgery (**A**). The score of (**A**) was 6.5 points by our evaluation. Only background-level staining was observed in the sham surgery group (**B**), which was assigned a score of 1.0.

The rise in the score was correlated with an ophthalmic evaluation of superficial punctate keratopathy (SPK). The corneal fluorescence pattern revealed severe SPK in the ELG plus ILG excised eyes compared with sham surgery eyes (Fig. 4). Compared with the ELG-only excision model (our previous study), a greater extent of corneal damage at each post-surgical time point was observed (Fig. 3).

### Severe inflammatory reaction was observed in the ELG plus ILG excised C57BL/6 WT mice

**Figure 5:**
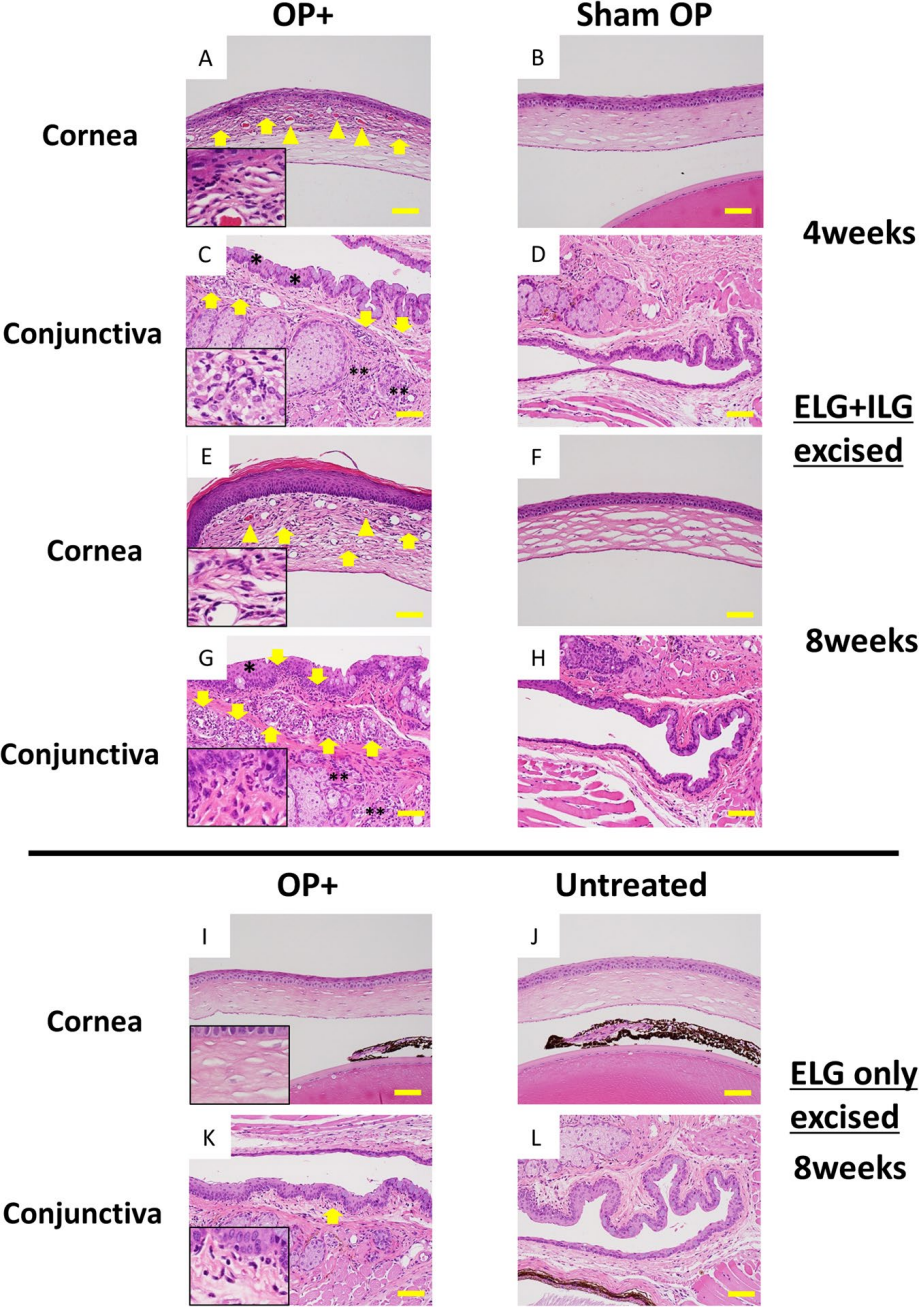
Histology of the ELG plus ILG excised C57BL/6 mice at 4- and 8-weeks after surgery, and ELG excised C57BL/6 mice at 8-weeks after surgery. Examination of the H&E-stained sections revealed significant severe inflammatory changes such as cell infiltration (**A**,**E**; yellow arrows), and neovascularization (**A**,**E**; yellow arrowheads) in the corneas of the ELG plus ILG excised mice at 4- and 8-weeks postoperatively. Moreover, significant inflammatory cell infiltration into the mucosal and submucosal layers (**C**,**G**; arrows), as well as conjunctival epithelial hyperplasia (**C**,**G**; asterisks), was observed in the conjunctiva of those mice. In particular, the palpebral conjunctival epithelium at 8-weeks postoperative (**G**; asterisk) was significantly thicker compared with that of sham-surgery control (**H**). Inflammatory cell infiltration was also observed in deeper area of the palpebral conjunctiva and around the meibomian gland (**C**,**G**; double asterisks). These histological changes became more advanced at 8 weeks (**E**,**G**) compared with 4 weeks postoperatively (**A**,**C**). No significant histological changes, compared with unoperated controls (not shown) were observed in the sham-surgery control eyes (**B**,**D**,**F**,**H**). In like manner, minimal inflammatory cell infiltration (*i*.*e*., categorized as a normal level) was observed in the submucosa of conjunctiva (**K**; arrow) in ELG only excised mice at 8-weeks after surgery, and no significant histological changes was observed in the cornea (**I**). In addition, no significant histological changes were observed in the untreated control eyes (**J**,**L**). Insertion to lower left of each figure (**A**,**C**,**E**,**G**,**I**,**K**) show 4 times magnification of original figures. Scale bar = 50 µm.

Examination of the Hematoxylin and Eosin (H&E)-stained sections of ocular surface tissues revealed severe inflammatory changes, including corneal epithelial damage (SPK), inflammatory cell infiltration into the epithelium and stroma, and neovascularization, in the corneas of the ELG plus ILG excised mice at 4, 8, and 12 weeks after surgery (Fig. 5 and Supplementary Fig. S1). Moreover, significant inflammatory cell infiltration into the mucosal and submucosal layers, as well as epithelial hyperplasia, was observed in the conjunctiva of those mice (Fig. 5 and Supplementary Fig. S1). In the eyelids, inflammatory cell infiltration was also observed in the vicinity of the meibomian glands (Fig. 5 and Supplementary Fig. S1). Those histological changes were advanced at 8-weeks after surgery compared with at 4- and 12-weeks after surgery using criteria for grading evaluation (Supplementary Fig. S1). No significant histological changes were observed in sham-surgery control eyes (Fig. 5). In addition, in agreement with our previous study results, inflammatory responses in the ocular surface of the ELG-only excised model were slight compared with those observed in the ELG plus ILG excised model (Fig. 5).

### Neutrophil infiltration was observed in the ocular surface of the ELG plus ILG excised LysM mice

**Figure 6:**
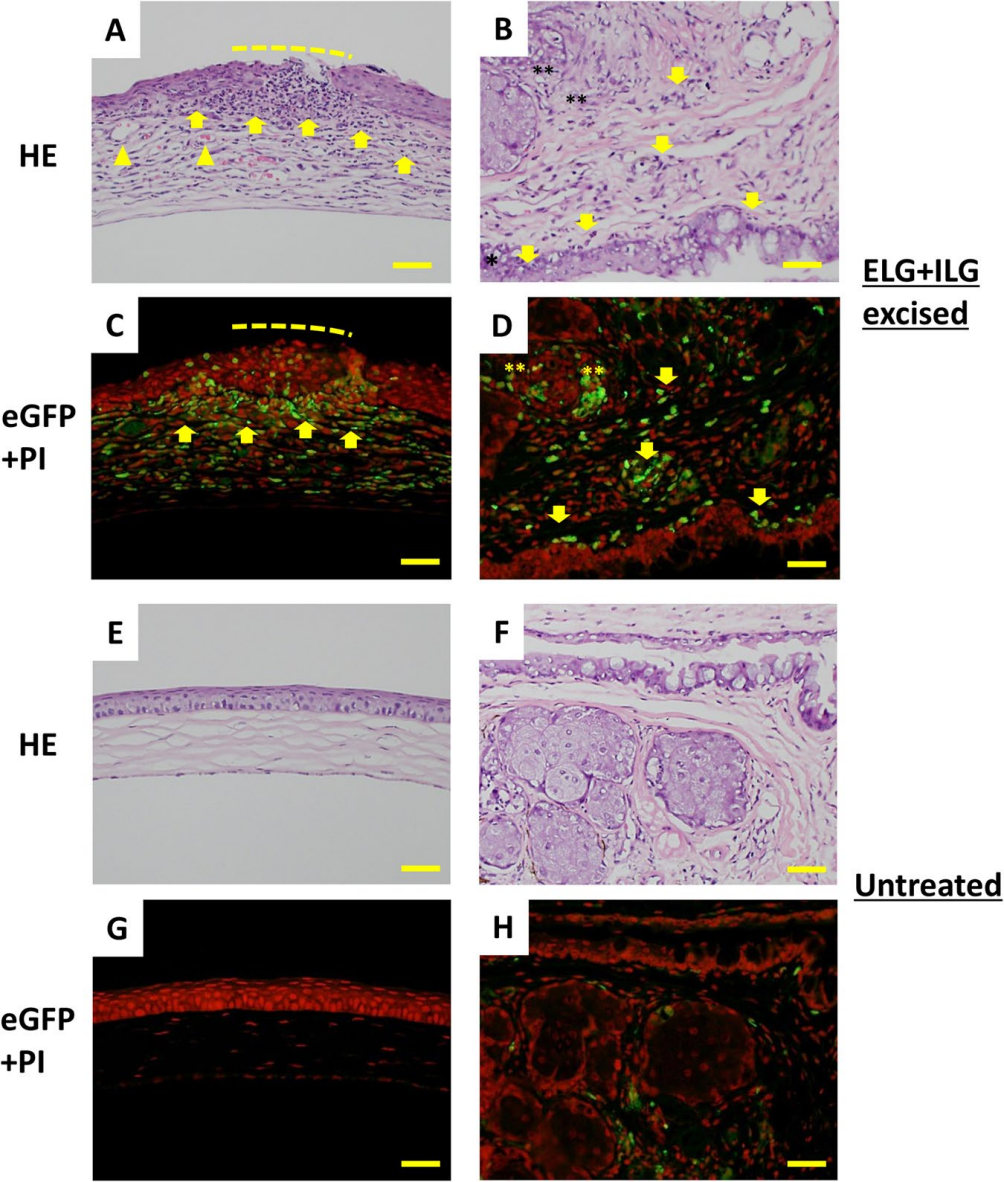
Histology of the ELG plus ILG excised LysM mice at 4-weeks after surgery. Examination of H&E-stained sections revealed severe inflammatory changes such as cell infiltration (**A**; arrows), neovascularization (**A**; arrowheads), and ulceration (**A**; dotted line) in the corneas of the ELG plus ILG excised mice. Moreover, inflammatory cell infiltration into the mucosal and submucosal layer (**B**; arrows), as well as conjunctival epithelial hyperplasia (**B**; asterisk), were observed in the conjunctiva of those mice. Inflammatory cell infiltration was also observed around the meibomian gland (**B**; double asterisks). In frozen sections, the main infiltrating cells detected in the ocular surface tissues were neutrophils, identified by a unique green fluorescence with intensity above background (**C**,**D**; arrow and double asterisks). The histological changes noted in operated animals were not observed in the eyes of untreated mice (**E**–**H**). Scale bar = 50 µm.

In otherwise unlabeled frozen sections, infiltration of fluorescent inflammatory cells was observed in the mucosal and submucosal layers of cornea and conjunctiva at 4-weeks after ELG plus ILG excision (Fig. 6). Inflammatory cell infiltration was also observed around and inside of the meibomian gland. On the basis of their morphology (e.g. multinuclear) in H&E-stained sections, and their specific fluorescence in the fluorescent observation, it was determined that these infiltrating cells in the ocular surface were predominantly neutrophils (Fig. 6). The histological appearance of these tissues was distinct from the normal histology observed in the eyes of untreated animals, and no infiltrating neutrophils were detected above normal levels (Fig. 6).

## Discussion

We document here that the morphological and physiological changes displayed in our new mouse dry eye model, generated by dual excision of both the ELG and ILG, are more severe than those observed in mice with excision of the ELG only (Figs 2 and 3). These changes, compared with the ELG-only excision model, consist of: 1) A more pronounced reduction in tear volume (by approximately 53%, Fig. 2), that was sustained without improvement during the entirety of the 10-week post-surgical observation period (Fig. 2); 2) A greater extent of corneal damage at each post-surgical time point (Fig. 3) -although in contrast to tear volume reduction, there was some improvement during the observation period (Figs 2); and 3) Persistent inflammatory changes in the cornea, conjunctiva, and meibomian gland, detected histologically even at 12 weeks after surgery (Shinomiya K *et al*., ARVO 2014 annual meeting, May 4, 2014, and Supplementary Fig. S1).

The changes documented here for ELG plus ILG excised mice are quite similar to those giving rise to the severe, chronic features of dry eye syndromes experienced by human patients, and they result from persistent and severe tear reduction. The tear volume reduction and consequent ocular surface changes of these mice were continuously observed, without improvement, even at 24-weeks after surgery in an additional follow-up study (data not shown).

Several methods for generating mouse models of dry eye have been previously reported, such as the topical administration of benzalkonium chloride (BAC)^32^, the injection of botulinum toxin into the lacrimal gland^33^, and breeding in a low-humidity and high-airflow environment combined with scopolamine hydrochloride administration^34^. However, many previously reported experimental dry eye models have the disadvantages of instability of the dry eye symptoms, a requirement for frequent administration of the therapeutic agent, the use of legally regulated toxic substances, and/or a required, specially arranged breeding environment. In contrast, our ELG plus ILG excised model will be more convenient and stable compared with existing animal models.

Judging from the neutrophil infiltration detected histologically, we theorize that the inflammatory changes on the ocular surface of the ELG plus ILG excised mouse model were induced secondarily by persistent severe tear volume reduction. Moreover, neutrophils were found to be the primary cell type infiltrating the ocular surface in this mouse model, thereby suggesting that a non-specific inflammatory reaction might occurr predominantly on the ocular surface of this model. The infiltration of lymphocytes or macrophages was infrequently observed, and we do not consider this to be a signature response in our model. In contrast to our findings, some previous reports using the desiccating stress mouse model indicate that lymphocyte infiltration in the ocular surface is dominant in the ocular surface tissue^35,36^. One possible explanation for this apparent discrepancy is that the two different dry eye models involve quite different causative and resultant pathophysiological mechanisms. Additional studies to characterize and differentiate these two models will be necessary to elucidate their differences at the tissue and organ level, specifically with respect to their inflammatory components.

However, the contribution of Th17 inflammatory responses might not be unexpected, because it is reported that neutrophils are frequently observed in Th17 inflammatory responses in mucosal tissues, and they also represent a first line of defense towards maintaining ocular surface homeostasis, and they can exert a regulatory role^37,38^.

It is apparent that the surgery for ELG plus ILG excision is more invasive and difficult than for an ELG only excision, particularly because the ILG of the mouse is located deep within the orbit. However we developed a minimally invasive approach for this protocol via the subcutaneous tissue of the temporal lid margin. The success rate of the surgery is estimated to be more than 90%, with experimental efficacy directly correlated to performance of the surgery. In addition, no side effects such as excessive bleeding, facioplegia, or disability of opening eyelids were observed during the observation period after surgery. An alternative animal model, the desiccating stress mouse model, is frequently used, and has recently attained the status of standard for the experimental dry eye model. However, the modified environment required for the breeding facility to maintain the dry eye status in the animals^34–36^ could be considered a drawback of this model. In contrast, the ELG plus ILG excision mouse model does not need a special environment for breeding. In addition, the ocular surface changes are observed continuously in our model, and to a great extent they do not improve in the absence of additional therapy or maintenance. In our estimation, the operational and functional characteristics of our model described here are advantageous, making this model more convenient than other dry eye mouse models^34^, including surgical (*e*.*g*., the ELG only excision model)^24,31^, for inducing severe tear volume reduction-type dry eye symptoms. It is important to note the possibility of using these different models for different proposes in accordance with specific investigative needs. For example, the desiccating stress model incorporates reversibility, and combines tear volume reduction with hyperevaporation. Nevertheless, the mouse model presented here appears to be convenient for inducing severe aqueous deficiency, as well as chronic and severe dry eye lesions on the ocular surface, enabling the development of new therapies for management and treatment.

## Methods

### Animals

Male LysM-eGFP^(+/−)^ (LysM) mice (n = 14, kindly gifted from Department of Immunology and Cell Biology, Graduate School of Medicine and Frontier Bioscience, Osaka University, Osaka, Japan), and C57BL/6 mice (female wild-type of LysM (n = 31); Crea Japan, Inc., Tokyo, Japan) were used for this study. The LysM mouse is a transgenic produced by insertion of the enhanced green fluorescent protein (eGFP) gene into the lysozyme M (lys) gene. This mouse shows specific green fluorescence derived from eGFP in myelo-monocytic lineage cells, especially neutrophils^39,40^.

This study was conducted in accordance with the ARVO Statement for the Use of Animals in Ophthalmic and Vision Research, and was approved by the Committee on Animal Research of Kyoto Prefectural University of Medicine. Animals were acclimated for at least 2 week before experimentation, were housed under conventional conditions (12-hour light/12-hour dark cycle, normal appropriate humidity and temperature under specific pathogen-free conditions), and were provided food and water *ad libitum*.

### Lacrimal Gland Excision

**Figure 7:**
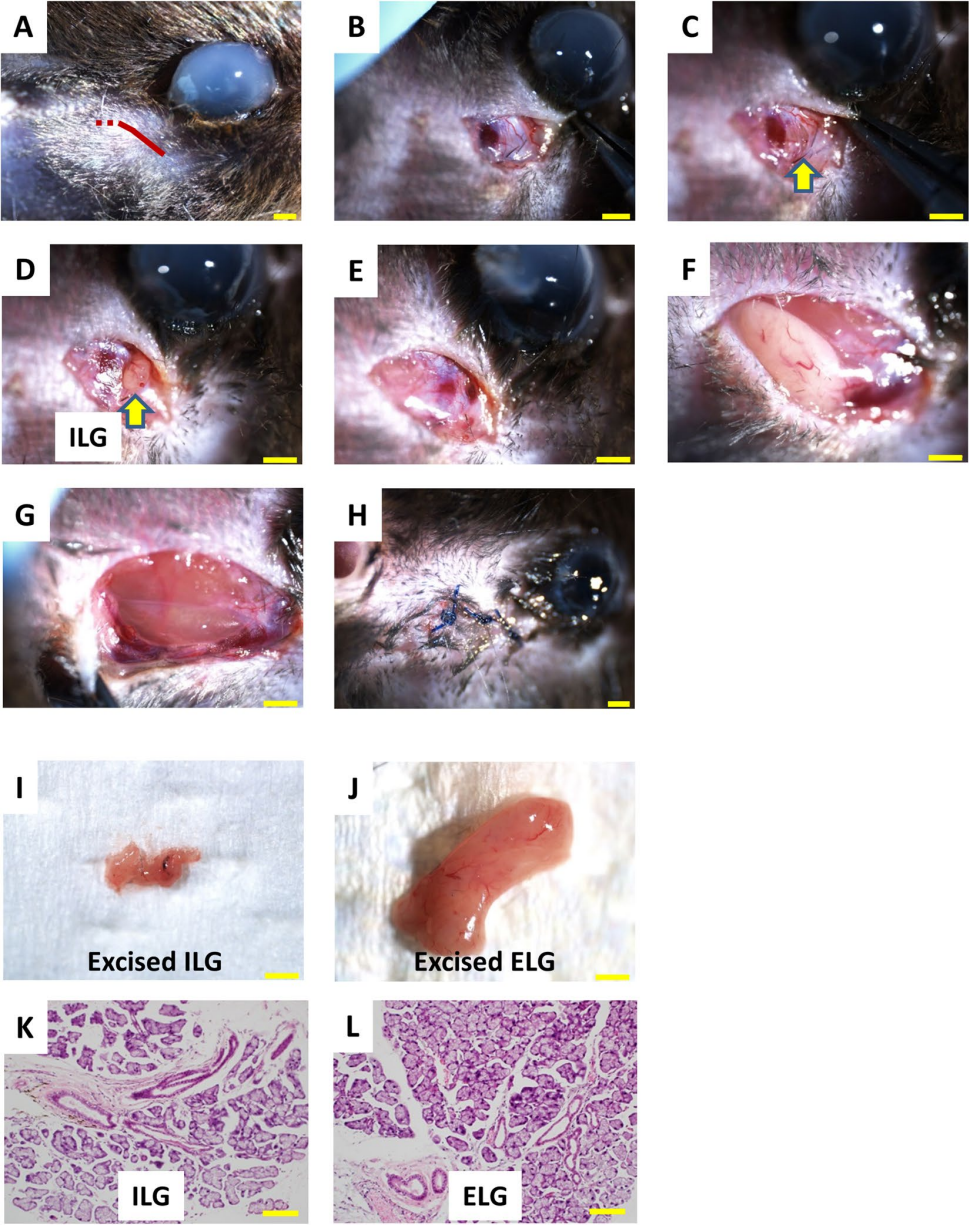
Images of the ELG plus ILG excisions recorded during surgery (**A**–**H**), showing the method of approach to the ILG via subcutaneous tissue, followed by excision of the ILG. An incision was made (**A**; red line) and the ILG was exposed (**B**–**D**; arrow). Next, ophthalmic forceps were used to carefully excise the ILG with the aid of a stereoscopic microscope (**D**,**E**; arrow). An additional incision was made anteriorly towards the ear (**A**; red dotted line), and the ELG was exposed (**F**) and excised (**F**,**G**). The incision was then sutured using 6-0 ophthalmic nylon thread, and ointment containing ofloxacin was applied (**H**). Macroscopic images of the excised ILG (**I**) and ELG (**J**). H&E staining of microscopic sections through the ILG (**K**) and ELG (**L**). Histologically, the structure of the excised ILG was characteristic of a typical exocrine gland. Scale bars = 1 mm in Figs **A**–**J**; = 100 μm in Figs **K** and **L**.

Animals were anesthetized by intraperitoneal administration of ketamine hydrochloride (Ketalar; Daiichi Sankyo Co., Ltd., Tokyo, Japan; 100 mg/kg) and xylazine hydrochloride (Celactal; Bayer Yakuhin Ltd., Osaka, Japan; 10 mg/kg). An incision was carefully made close to the temporal lid margin to expose the ILG, and the ILG was excised from the orbit using ophthalmic forceps with the aid of a stereoscopic microscope. Next, an additional incision was made anterior to the ear, and the ELG was exposed and then excised. The surgical incisions were then sutured using 6-0 ophthalmic nylon thread, and ointment containing ofloxacin (Tarivid Ophthalmic Ointment 0.3%; Santen Pharmaceutical Co., Ltd., Osaka, Japan) was applied. The ELG plus ILG excisions were made only on the right side, and the left side underwent either a sham surgery (C57BL/6 wild type mice) or no surgical treatment (LysM mice) (Fig. 7).

### Ophthalmological Examination

To evaluate dry eye symptoms, we assessed corneal surface integrity after application of fluorescein, and also measured tear production, at 2 weeks before surgery and at 2, 4, 6, 8, and 10 weeks after surgery.

#### Corneal fluorescein infiltration

Animals were anesthetized by intraperitoneal administration of ketamine and xylazine as described above. Fluorescein working solution was formulated by extracting one Fluores Ocular Examination Test Paper (Showa-Yakuhin-Kako Co., Ltd., Tokyo, Japan) in 300 µL of sterile saline, resulting in an approximately 2.3 mg/mL solution of fluorescein sodium salt. After approximately 10 µL of fluorescein solution was applied to both eyes, they were rinsed with saline and observed using a blue-free filtered light source and digital camera (Nikon D7000; Nikon Corporation, Tokyo, Japan). Digital images of the corneal surfaces were captured, and the fluorescein infiltration patterns were scored, using a modification of the method of Murakami *et al*. (Fig. 3)^41^. The fluorescein infiltration score was categorized from 0 to 3 (0 = no fluorescence, 1 = fluorescence resembling sparse dots, 2 = dense dot-like pattern, 3 = very dense dot-like fluorescence) depending on the observation, with determinations made for each 1/3 of the cornea (upper, intermediate, and lower). Infiltration patterns intermediate between 0 and 1, 1 and 2, 2 and 3 were categorized as 0.5, 1.5, or 2.5, respectively. Finally, these scores were summed to provide a composite score for each eye (minimum = 0, and maximum = 9).

#### Tear volume measurement

Tear production of each eye was measured twice by use of cotton thread containing phenol red (Zone-Quick; Showa Yakuhin Kako Co., Ltd.) and averaged. Animals were fixed manually without anesthesia, the cotton threads were inserted into the center of the lower fornix for 15 seconds, and then the length of wet portion of the thread was measured immediately. The length of wet portion of the thread was converted to the corresponding secreted tear volume using an internally validated standard curve.

### Histological Analysis

#### C57BL/6 WT mice samples for hematoxylin-eosin staining

At 4, 8, and 12 weeks after surgery, animals were anesthetized by intraperitoneal administration of ketamine and xylazine, and sacrificed immediately by cutting the descending aorta and inferior vena cava. Whole eyes containing upper and lower eyelids were resected, and fixed by immersion with 10% neutral buffered formalin (Nacalai Tesque Inc., Kyoto, Japan) for about 1 week. Then, the tissues were embedded in paraffin using standard procedures. Approximately 3-µm thickness serial sections were cut using a sliding microtome (ROM-380; Yamato Kohki Industrial Co., Ltd., Saitama, Japan), placed on glass slides (Fine Frost Micro Slide Grass; Matsunami Glass Ind., Ltd., Osaka, Japan). The sections were deparaffinized and rehydrated, and then stained with hematoxylin-eosin (H&E) using standard procedures.

#### LysM mice samples for fluorescent observation

At 4 weeks after surgery, whole eyes containing upper and lower eyelids were resected by the same methods as mentioned previously, fixed by immersion with 10% neutral buffered formalin for 1 day, rinsed successively in phosphate buffered saline (PBS) containing 10% and 20% sucrose, and finally embedded in Tissue Tek O.C.T. compound (Sakura Finetek Japan Co., Ltd., Tokyo, Japan) and snap frozen in liquid nitrogen. Approximately 5-µm thickness serial sections, cut using a cryostat (CM3050S; Leica Biosystems Nussloch GmbH., Nussloch, Germany), were placed on aminosilane-coated glass slides (Dako Silanized Slide; Dako Denmark A/S, Glostrup, Denmark), air dried. The serial sections were rinsed with PBS to remove the OCT compound, and then coverslips were mounted with VECTASHIELD Mounting Medium containing propidium iodide (Vector Laboratories, Inc., Burlingame, CA, USA).

### Analysis

The statistical significance of the results of fluorescein infiltration and tear volume measurement was determined using the unpaired Student’s t-test or Welch’s t-test, and a P-value < 0.05 was considered statistically significant.

H&E stained sections were observed by use of a light microscope (BX-63; Olympus Corporation, Tokyo, Japan) to assess histological changes in the cornea and the conjunctiva. Digital images of the sections were recorded by use of a CCD camera (DP72; Olympus Corporation). The histological findings were then scored from 0 to 4 (0 = no findings, 1 = slight change, 2 = mild change, 3 = moderate change, 4 = severe change) depending on the grade of the lesion.

The unlabeled sections of LysM mice were also observed by fluorescence microscopy without additional staining to determine neutrophil infiltration.

The statistical significance of the scores of the histological findings was determined using the unpaired Student’s t-test and a P < 0.05 was considered statistically significant.

### Data Availability

The datasets generated during and/or analyzed during the current study are available from the corresponding author in response to reasonable request.

## Electronic supplementary material

Supplementary information
