# Supplementary material for: A new dry eye mouse model produced by exorbital and intraorbital lacrimal gland excision
Source: Sci Rep. 2018 Jan 24;8:1483. doi: 10.1038/s41598-018-19578-6 (PMC5784089; doi:10.1038/s41598-018-19578-6)
Supplement: Supplementary file 1 — Supplementary information [file 41598_2018_19578_MOESM1_ESM.pdf]

A new dry eye mouse model produced by exorbital and intraorbital lacrimal gland excision

*Katsuhiko Shinomiya[1, 2], Mayumi Ueta[3], and Shigeru Kinoshita[3]

FIGURE LEGENDS

Supplementary figure S1. Summary of the histological change of the cornea, conjunctiva, and meibomian gland. The histological findings for cornea, conjunctiva and Meibomian gland were scored from 0 to 4 (0=no findings, 1=slight change, 2=mild change, 3=moderate change 4=severe change) depending on the grade of the lesion. The findings for cornea were classified with respect to epithelial damage, neovascularization, inflammatory cell infiltration, and granulomatous change. The findings for conjunctiva were evaluated with respect to intra-epithelial and submucosal inflammatory cell infiltration, epithelial hypertrophy, and submucosal edematous change. The findings for meibomian gland were classified with respect to inflammatory cell infiltration into the acinar and stromal regions. For each tissue the additive scores for each animal were computed. The histological scores for the cornea, conjunctiva and meibomian gland of

the ELG plus ILG excised mice were significantly increased compared with those for sham surgery mice. These histological scores showed the highest statistical significance, compared to controls, at 8 weeks following surgery. Each data point represents mean ± SEM. * and *** indicate statistical significance ($P<0.05$ and 0.001, respectively) compared with sham OP by unpaired Student's t-test or Welch's t-test. #, ## and ### indicate statistical significance ($P<0.05$, 0.01 and 0.001, respectively) compared with each data by unpaired Student's t-test or Welch's t-test. NS indicates no statistical significance between data points.

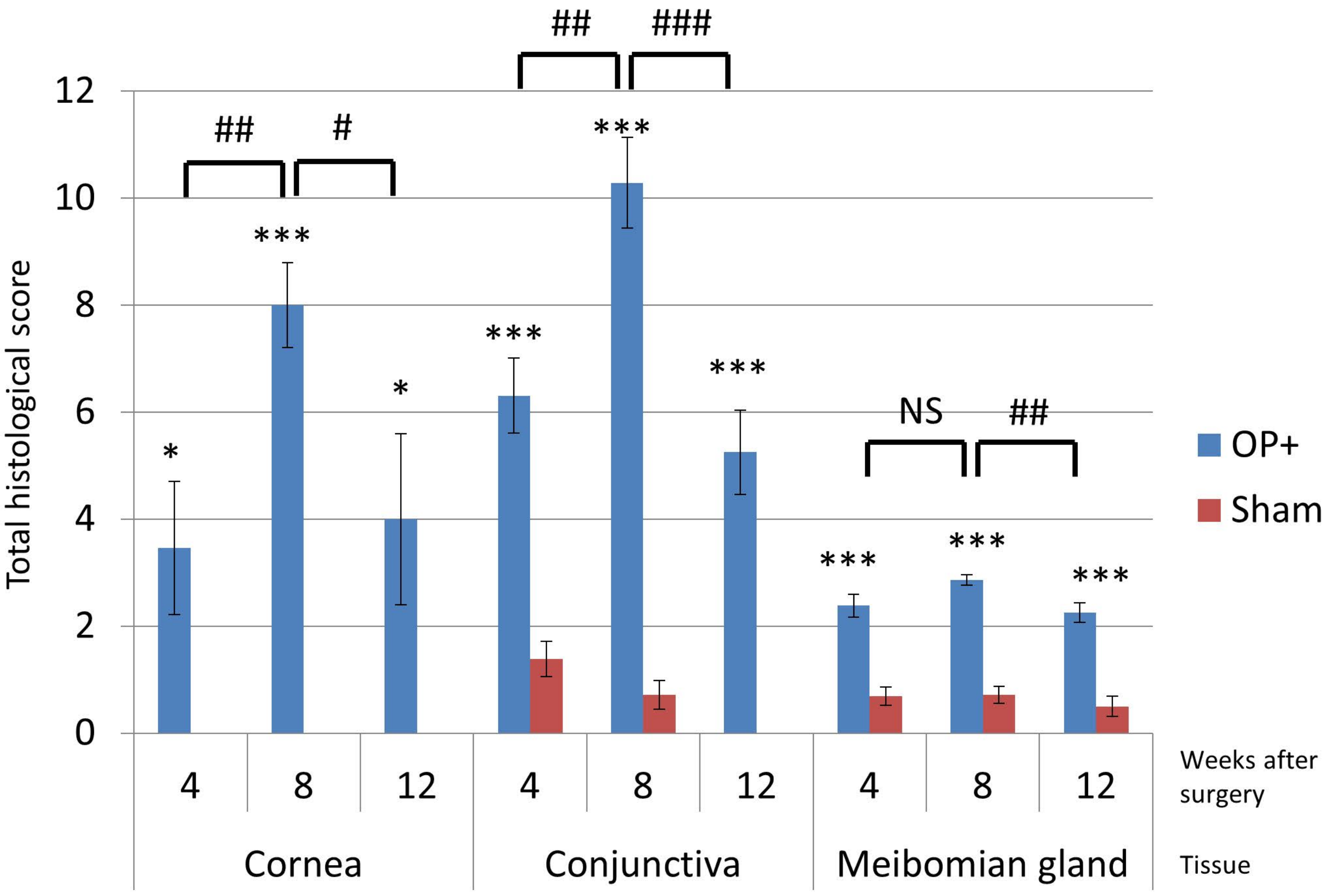

Supplementary figure S1.
